# Between-Population Outbreeding Affects Plant Defence

**DOI:** 10.1371/journal.pone.0012614

**Published:** 2010-09-07

**Authors:** Roosa Leimu, Markus Fischer

**Affiliations:** 1 Institute for Biochemistry and Biology, University of Potsdam, Potsdam, Germany; 2 Institute of Plant Sciences and Oeschger Centre, and Botanical Garden, University of Bern, Bern, Switzerland; University Copenhagen, Denmark

## Abstract

Between-population crosses may replenish genetic variation of populations, but may also result in outbreeding depression. Apart from direct effects on plant fitness, these outbreeding effects can also alter plant-herbivore interactions by influencing plant tolerance and resistance to herbivory. We investigated effects of experimental within- and between-population outbreeding on herbivore resistance, tolerance and plant fitness using plants from 13 to 19 *Lychnis flos-cuculi* populations. We found no evidence for outbreeding depression in resistance reflected by the amount of leaf area consumed. However, herbivore performance was greater when fed on plants from between-population compared to within-population crosses. This can reflect outbreeding depression in resistance and/or outbreeding effects on plant quality for the herbivores. The effects of type of cross on the relationship between herbivore damage and plant fitness varied among populations. This demonstrates how between-population outbreeding effects on tolerance range from outbreeding depression to outbreeding benefits among plant populations. Finally, herbivore damage strengthened the observed outbreeding effects on plant fitness in several populations. These results raise novel considerations on the impact of outbreeding on the joint evolution of resistance and tolerance, and on the evolution of multiple defence strategies.

## Introduction

Inter-population crosses and transplantations of plants among populations are used to restore genetically eroded populations [1], [2] despite the fact that this can result in outbreeding depression, especially in fragmented, and thus genetically differentiated, plant populations (e.g., [2]–[5]). Outbreeding depression can arise because of breaking up co-adapted gene complexes [6], or due to the disruption of local adaptation, which is common at least in large plant populations [7]. Several mechanisms may contribute to the degree of expressed outbreeding depression depending on the effects of the genetic and environmental histories of populations, in particular on the interplay between selection, drift, gene flow and inbreeding [8]. Thus, outbreeding effects are likely to vary among populations. Compared to large continuous populations, fragmented populations may experience lower outbreeding depression following inter-population crosses due to positive outbreeding effects on heterozygosity. Alternatively, due to strong directional selection, populations with low levels of genetic variation can actually be more strongly adapted to local conditions and hence more likely to suffer from outbreeding depression following crosses between distinct populations.

In addition to direct negative effects on plant fitness due to reduced genetic variation and increased inbreeding (reviewed in [9]), habitat fragmentation can influence plant fitness indirectly by modifying interactions with other species. Fragmentation alters the abundance and composition of communities of natural enemies of plants [10], [11]. Such changes can alter both selective pressures exerted by herbivores and abilities of plants to respond. Several studies have found inbreeding depression in herbivore resistance [12]–[15] and tolerance [16] whereas outbreeding effects on these plant defence strategies are much less studied, especially in the fragmentation context. Studies examining effects of hybridization between species on plant resistance against natural enemies have either reported no differences between hybrids and parental plants, an additive effect, hybrid susceptibility or dominance of the susceptible parent (reviewed in [17]).

Of the two general plant defence strategies against herbivores, resistance refers to any plant trait that influences the amount of damage while tolerance reflects the degree to which a plant can re-grow and reproduce after damage [18]–[21]. Like fitness, resistance and tolerance are complex traits often composed of several underlying characters or mechanisms [22]. Changes in genetic variation due to inbreeding and outbreeding are likely to modify plant defence strategies against herbivores, because these strategies often have a genetic basis (e.g., [23], [24]). It is likely that outbreeding effects on resistance and tolerance influence outbreeding effects on fitness. We hypothesize that if there is outbreeding depression in herbivore resistance and/or tolerance this should result in greater outbreeding depression in fitness of plants damaged by herbivores compared to undamaged plants. This importantly implies that outbreeding depression might have been underestimated in previous investigations of the consequences of between-population crosses, because only undamaged plants were considered in crossing experiments. Moreover, if outbreeding affects resistance and tolerance differently, this may influence their joint evolution.

Here we investigated outbreeding effects on herbivore resistance against the generalist herbivore *Arianta arbustorum*, tolerance to snail damage and artificial damage (clipping), and plant fitness in greenhouse with plants originating from 13 to 19 (depending on the experiment) fragmented *Lychnis flos-cuculi* populations. These populations are known to suffer from negative genetic effects of habitat fragmentation as genetic variation within population is reduced and inbreeding increased in small populations [25]–[28], and the populations are genetically differentiated from each other [27], [28]. Moreover, the populations occur in habitats that differ in the levels of herbivore damage and in abiotic conditions [27], [29], [30]. A reciprocal transplant experiment involving the same plant populations found adaptation to ecological conditions [30] suggesting detrimental effects of between-population outcrossing for offspring fitness. In our greenhouse experiments, we used F2 plants from two generations of experimental outcrosses within and between populations.

We addressed the following questions: 1) Are plants from between-population crosses less resistant and tolerant against herbivory than plants from within-population crosses? 2) Do outbreeding effects on resistance and on tolerance vary among plant populations? 3) Does plant damage modify outbreeding effects on plant fitness?

## Results

### Outbreeding effects on plant resistance and tolerance

**Figure 1:**
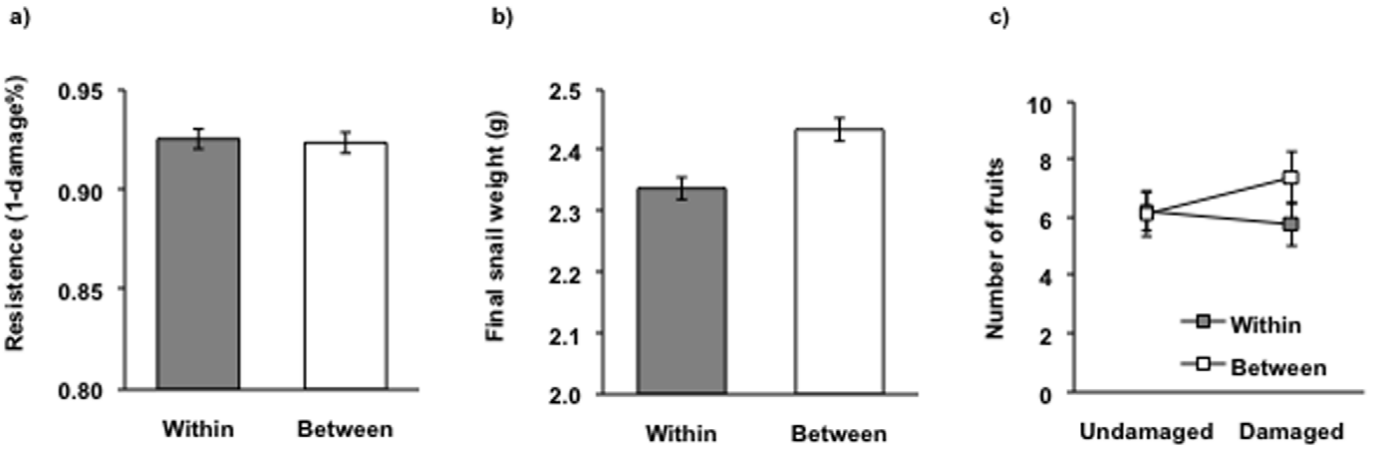
Mean effects of outbreeding on resistance and tolerance. Average resistance, measured as a) 1- proportion of damaged leaf area, b) final snail mass in the snail herbivory experiment with plants resulting from two generations of within- and between-population crosses of plants from 13 populations of *Lychnis flos-cuculi*. c) Effects of outbreeding on tolerance to clipping in the clipping experiment with plants resulting from two generations of within- and between-population crosses of plants from 19 populations of *Lychnis flos-cuculi*. Average effects of clipping on number of fruits produced after damage (1c). Covariate-adjusted least-squares means estimates and estimated standard errors are presented.

Resistance, measured as 1-the proportion of leaf area damaged by snails, did not differ significantly between plants from within- and between-population crosses (Table 1, Fig. 1a). On average, snails grew larger when fed on plants from between-population crosses compared to plants from within-population crosses indicating lower resistance of plants from between-population crosses, and therefore outbreeding depression (Table 1, Fig. 1b). Alternatively, the results might also reflect impacts of outbreeding on plant quality for the herbivores.

**Table 1:** Outbreeding effects on resistance in 13 populations of *Lychnis flos-cuculi*.

	Source	DF	F	P
1-Proportion of leaf area damaged	Number of rosette leaves	1	29.35	0.0001
	Initial snail mass	1	4.27	0.0396
	Population	12	1.75	0.0548
	Cross	1	0.09	0.7627
	Population × Cross	11	1.16	0.3132
	Family (Population)	121	1.16	0.0848
	Residual	356		
Final snail mass	Initial snail mass	1	1596.21	0.0001
	Population	12	0.72	0.7317
	Cross	1	8.32	0.0042
	Population × Cross	11	1.62	0.0924
	Family (Population)	121	1.01	0.4582
	Residual	344		

ANCOVA summary of effects of populations, cross and family on two measures of resistance to snail herbivory (1-proportion of leaf damaged; snail performance) in our snail herbivory experiment with plants resulting from two generations of within- and between-population crosses of plants from 13 populations of *Lychnis flos-cuculi*.

**Figure 2:**
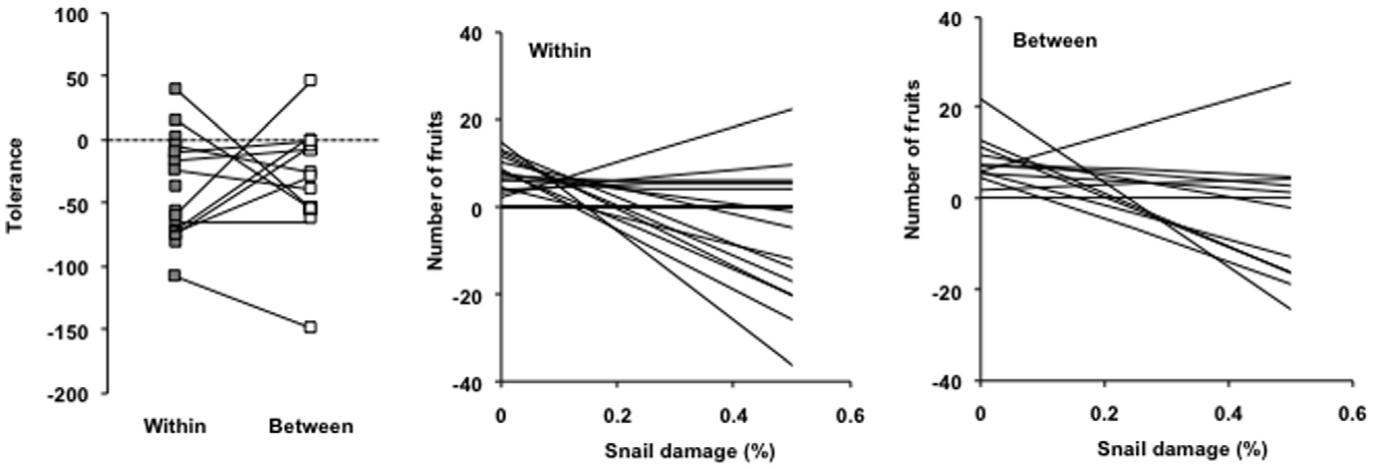
Among-population variation in outbreeding effects on tolerance and plant fitness. Among-population variation in a) outbreeding effects on tolerance and in the effects of snail damage on the number of fruits produced for b) plants from within-population and c) between-population crosses in the snail herbivory experiment. Tolerance is assessed as slope of fitness (number of fruits) and leaf damage %. Thus, tolerance is given in units of change in the number of fruits per % leaf area damaged. The squares in a) denote slopes for plants from within- (grey symbols) and between-population (white symbols) crosses for each population. The lines illustrate differences in tolerance between plants within-and between population crosses for each population. Tolerance >0 indicates overcompensation, tolerance  = 0 full compensation, and tolerance <0 undercompensation. In figures b) and c) each line represents a population.

Outbreeding had no significant effects on tolerance to artificial damage (Table 2; Fig. 1c). Snail damage significantly reduced the number of fruits produced (Table 2) indicating poor tolerance and under-compensation (mean tolerance <0 in Fig. 2a).

**Table 2:** Outbreeding effects on tolerance to snail and artificial damage.

	*Source*	*DF*	*F*	*P*
***a) Artificial damage***	Initial # leaves	1	34.65	0.0001
Number of Fruits	Population	18	1.61	0.0526
	Cross	1	1.75	0.1859
	Artificial damage	1	1.00	0.3172
	Family(Population)	129	2.48	0.0001
	Population × Cross	11	1.14	0.3278
	Population × Artificial damage	17	0.61	0.8827
	Artificial damage × Cross	1	1.58	0.2088
	Population × Cross × Artificial damage	11	1.13	0.3367
	Artificial damage × Family(Population)	128	0.81	0.9350
	Residual	757		
***b) Snail damage***	Initial # leaves	1	19.67	0.0001
Number of Fruits	Population	18	2.92	0.0001
	Cross	1	0.18	0.6734
	Family(population)	131	2.51	0.0001
	Damage by snails (%)	1	11.42	0.0008
	Population × Cross	11	1.89	0.0377
	Population × Damage by snails (%)	18	1.98	0.0377
	Cross × Damage by snails (%)	1	0.26	0.6072
	Population × Cross × Damage by snails (%)	11	2.37	0.0070
	Family(population) × Damage by snails (%)	129	1.08	0.2807
	Residual	809		

ANCOVA summary of a) effects of populations, cross (with or between population outbreeding) and artificial damage (clipped and sprayed versus undamaged) on the number fruits produced after damage in our artificial-damage experiment and b) effects of populations, cross, family and level of snail damage on the number of fruits produced after damage in our snail herbivory experiment.

### Among-population variation in outbreeding effects on resistance and tolerance

Tolerance to snail damage varied among populations as indicated by the significant population × damage level interaction for the number of fruits produced (Table 2). Moreover, the significant interaction of cross, population and damage level indicates that the slope of the relationship between damage and fruit production was influenced by population and cross, i.e., the effects of outbreeding on tolerance varied among populations (Table 2, Fig. 2). The average tolerance estimates for each population to snail damage of plants from within- and between population outcrosses were not correlated (*r* = −0.035, *P* = 0.914 *N* = 19). This indicates that outbreeding affects tolerance differently in different populations and, therefore, the impact of outbreeding on tolerance is not predictable. Outbreeding effects on resistance, or tolerance to clipping did not vary among populations (Table 1,Table 2).

### Impact of plant damage on outbreeding effects on fitness

Snail damage influenced outbreeding effects on plant fitness (number of fruits; significant population×cross×damage interaction, Table 2): these outbreeding effects varied among populations from outbreeding benefits to outbreeding depression depending on the level of damage by the snails (Fig 2 b, c).

When the bagged control plants were analyzed separately, we did not find any significant differences in the number of fruits produced between plants from within- and between-population crosses (*F* = 0.33, *df* = 1, *p* = 0.5677). The population×cross interaction was also not significant (*F* = 1.47, *df* = 11, *p* = 0.1385). These results suggest no outbreeding depression or benefits in the fitness of undamaged plants. These findings and those on outbreeding effects on plant fitness in relation to snail damage suggest that outbreeding depression in fitness may only become apparent under herbivore damage. Artificial damage did not influence plant fitness nor did it modify the observed outbreeding effects on plant fitness (Table 2; Fig 1 d, e).

## Discussion

### Outbreeding effects on resistance and tolerance

The amount of leaf area consumed by the snails did not differ between plants from within- and between-population crosses. This suggests lack of outbreeding depression in resistance. However, the snails grew larger when fed on plants from between-population crosses compared to when feeding on within-population crosses. This can be interpreted as outbreeding depression in resistance and/or outbreeding effects on plant quality for the herbivores.

What are the potential mechanisms that can result in outbreeding depression in resistance? Due to the additive inheritance of resistance between-population outcrossing could result in either outbreeding depression in resistance or hybrid vigour, that is, greater resistance of the resulting offspring, depending on the phenotypic means of the populations in question [17], [31]. If plants from a population with low resistance were crossed with plants from a population with high resistance, this would result in offspring with intermediate levels of resistance between two parental origins [17], [31]. Moreover, compared to the maternal plants from the population with lower resistance, the offspring would benefit from between-population outbreeding. On the other hand, if plants from a population with high resistance were crossed with those from a low resistance, the offspring would suffer from outbreeding depression [17], [31]. Likewise, the effect of dominance may appear either as outbreeding depression or as hybrid vigour, depending on the reference population. Finally, outbreeding between plants from distant populations can lead to inferior resistance due to disrupted interactions among loci that reflect epistatic gene actions [32], [33] or local adaptation [3], or due to formation of unfavourable epistatic interactions or underdominance [34]. In nature pollen and seed dispersal often occur among several populations, and likewise mixing of pollen donor and seed origins is also commonly considered in restoration. Therefore, to obtain a general assessment of outbreeding effects requires mixing of outbreeding origins, that is crossing each target population with pollen from several donor populations, as was done in our study.

Our findings are in line with previous studies demonstrating that inbreeding and outbreeding effects on resistance vary depending on how resistance is determined (e.g., [7], [35]). Using damage levels or herbivore performance as indicators of plant resistance is likely to reflect responses of a number of underlying traits that can be influenced differently by inbreeding or outbreeding, thus resulting in the observed variation [35]. Moreover, these measures do not allow accurately disentangling effects on plant quality and defence *per se*. For example, instead of or in addition to altering resistance, crossing plants between populations may increase the nutritive quality of the plants for herbivores. Plants are, in general, poor food sources for herbivores due to their low nutritive quality [36], [37]. Generalist herbivores are often more strongly affected by plant defence than by plant quality [38]. Therefore, the better snail performance on plants from between population crosses is more likely to arise due to outbreeding effects on defence (i.e., reduced defence) rather than quality. Moreover, because the plants in our experiment were grown in rich soil and under optimal growth conditions, it is less likely that resources would limit growth or reproduction and, therefore, constrain allocation to defence (sensu [39]).

The plant stress hypothesis predicts stressed plants to be beneficial for herbivores [40]. Hull-Sanders and Eubanks [41] applied the plant stress hypothesis [40] to hypothesize impacts of inbreeding and outbreeding on plant resistance and levels of herbivory, and predicted varying impacts on specialist and generalist herbivores. As pointed out above, generalist herbivores are predicted to be affected by plant defence rather than plant quality and, hence, should perform better on stressed and therefore poorly defended plants [40], such as inbred plants, as was found by Hull-Sanders and Eubanks [41]. Likewise, if between-population outbreeding resulted in stressed and therefore less defended plants, snail growth should be greater on plants from between-population compared to within-population crosses as was observed in our experiment. The absence of any fitness differences in undamaged plants from within- versus between-population crosses indicates that any stress caused by between-population outbreeding is certainly not expressed directly in terms of reduced fitness under undamaged conditions. However, the increased snail performance on plants from between-population crosses suggests that these plants might be more stressed in terms of having lower defence, which, in turn, might cause indirect negative effects on plant fitness via reduced impact on herbivores as we discuss further below. Alternatively, the better snail performance on plants from between-population crosses might be explained by increased nutritive quality of these plants. As our goal was the general test of outbreeding by herbivory interactions, distinguishing between outbreeding effects on defence and nutritive quality is unfortunately beyond the scope of the current paper.

The observed effects of outbreeding on tolerance were comprised of positive, neutral and negative effects depending on the type of damage. Tolerance and compensatory responses can vary depending on types of herbivores and the type of damage they cause, because these may be associated with different mechanisms of tolerance [42], [43]. Plants are often differently tolerant to artificial and natural damage, because these damages may pose different types of stress for the plants and, therefore, result in different responses in the plants (e.g., [21], [44]). Controversy exists over which of the two should be used in experimentation (e.g., [21], [44]). Our results add to the notion that tolerance may differ depending on the type of damage, and that estimates of tolerance to artificial damage should not be directly interpreted as tolerance against damage by herbivores. The exceptionally high tolerance to artificial damage and the relatively good tolerance to snail damage might be explained by the experimental greenhouse conditions, which reflected optimal growth conditions for *Lychnis flos-cuculi*. The temperature, moist, nutrient and light conditions mimicked those of mesic open wet grassland habitats where *L. flos-cuculi* preferably grows in nature. In addition to the optimal biotic conditions, the plants grew without competition in the greenhouse, which might have further increased their ability to compensate damage compared to natural field conditions. Furthermore, although tolerance is likely to be costly (e.g., [21]), such costs may not be apparent under the favourable greenhouse conditions, or may only manifest themselves in the following season in terms of reduced growth and/or reproduction or both.

Our findings suggest that outbreeding affects the different defence strategies (resistance and tolerance) and their components differently. This raises novel considerations on the impact of outbreeding on the joint evolution of resistance and tolerance. A trade-off between resistance and tolerance is often expected, although so far the evidence supporting such a trade-off is weak [45], [46]. It has been acknowledged that whether a trade-off exists between tolerance and resistance depends on environmental conditions [45]–[47]. Our study was not designed for an accurate analysis of such trade-offs by investigating genetic correlations between resistance and tolerance, which would require a higher number of replicates of the families in each treatment. Our study, however, suggests that the level of outbreeding may influence resistance-tolerance trade-offs. This could be the case, if tolerance and resistance of plant families were differently influenced by outbreeding, that is, families with positive effects of among-population outbreeding on tolerance show negative effects on resistance and vice versa. At the population level, outbreeding could affect these trade-offs, because among-population outbreeding increased resistance (measured in terms of snail size) in some of the populations whereas in others it was found to influence tolerance.

### Among-population variation in outbreeding effects on resistance and tolerance

The observed variation among populations in effects of inter-population outbreeding on tolerance, and a tendency for such variation in plant resistance in terms of snail performance, could arise due to among-population variation in abiotic and biotic factors and genetic history (e.g., [15], [46]). Inter-population outbreeding can be beneficial for highly inbred plant populations, because such outbreeding is likely to enhance offspring fitness [48], [49], and may also result in increased herbivore resistance and tolerance. However, crossing plants originating from populations each with adaptations to their local conditions may, in turn, result in reduced fitness and herbivore defence, if these local adaptations break due to outbreeding [2], [3], [5], [6]. In addition to inbreeding history, genetic differentiation of populations and their geographic distance can affect outbreeding depression: crossing genetically and geographically distant populations is likely to result in outbreeding depression whereas crossing genetically similar and nearby populations will not [1]. The studied populations are genetically differentiated from each other [27], [28], but neither genetic nor geographic distance between populations affected plant performance in a reciprocal replant-transplant field experiment involving 15 populations of *L. flos-cuculi*, including some of our study populations [30]. Therefore, it is unlikely that the observed among-populations variation in outbreeding effects on tolerance is explained by genetic or geographic distance among populations. This variation in outbreeding effects on tolerance could reflect among-population variation in the underlying mechanisms of tolerance and/or in related allocation patterns (e.g., [21]). It also suggests that several mechanisms might be responsible for tolerance, and that these mechanisms are likely to be differently influenced by outbreeding. Patterns of resource allocation between different functions, such as between growth and reproduction, prior or after damage may result in varying levels of tolerance observed in different plant traits [21], which could further contribute to the observed variation in outbreeding effects on tolerance to snail damage. The lack of correlation between plants from within- and between-population crosses in tolerance to snail damage suggests that the impact of outbreeding on tolerance varies among populations and, therefore, is not predictable. In any case, the fact that outbreeding effects on tolerance and resistance can vary among populations ranging from outbreeding benefits to depression highlights the importance of considering multiple populations when investigating outbreeding depression.

### Impact of plant damage on outbreeding effects on fitness

We initially predicted that outbreeding depression in herbivore resistance and/or tolerance or both should result in greater outbreeding depression in fitness of damaged plants compared to undamaged plants. In line with our prediction, outbreeding depression in fitness was absent for undamaged plants, but apparent in plants damaged by the snails, though this was the case only for some of the populations.

The fact that outbreeding effects on plant fitness under herbivore damage varied from positive to negative among populations indicates that inter-population crosses presumably influenced both plant quality and defence, and the related resource allocation patterns. Disentangling these different effects of outbreeding requires future studies. Our results, however, strongly suggest that previous studies investigating the consequences of between-population crosses may have underestimated outbreeding depression by only considering undamaged plants.

### Conclusions

The finding that inter-population outbreeding affects herbivore resistance and tolerance differently, and that these effects differ depending on type of damage, raise novel considerations on the impact of outbreeding on the joint evolution of resistance and tolerance, and on the evolution of multiple defence strategies. Understanding how different genetic configurations influence the evolution of plant-herbivore interactions is essential if we are to understand how the genetic consequences of habitat fragmentation influence species interactions and ultimately the structure of communities. Hence, the role of mating systems, and the levels of inbreeding and outbreeding, should be more rigorously investigated in future studies on the evolution of resistance and tolerance. Furthermore, because outbreeding effects on tolerance and resistance are likely to vary among populations, conclusions on outbreeding effects on these defence strategies should not be drawn from studies considering only one or very few populations.

Our results also highlight how estimates of outbreeding depression or outbreeding benefits on plant fitness from experimental conditions, where biotic or abiotic stress are not considered, can represent biased estimates of outbreeding effects under natural conditions. Finally, our findings have important implications for conservation. Clearly, plant-herbivore interactions should not be neglected and different plant defence strategies should be considered before mixing gene pools or transplanting plants among populations for conservation purposes.

## Materials and Methods

### Study species

The ragged robin, *Lychnis flos-cuculi* (Caryophyllaceae), is a polycarpic perennial herb that occurs in sunny and moist habitats including wet hay meadows and calcareous fens. It is still widespread and abundant throughout its distribution range, but its populations have become smaller and more isolated in recent decades due to the loss and fragmentation of suitable habitats.

*Lychnis flos-cuculi* is self-compatible, but its flowers are considered to be predominantly outcrossed [27], [28], [50]. Plants grow up to 70 cm tall flowering stems from leaf rosettes, and produce up to 60 insect-pollinated flowers. A number of generalist herbivores including snails, leaf miners and lepidopteran larvae [51], and some specialists [29] have been observed to attack the plants in the field. Levels of herbivory vary significantly among populations and years. In a study conducted in Switzerland, average damage levels were found to vary between populations ranging from 3% to 74% of leaves damaged [29]. Moreover, damage levels have been found to vary among maternal plant families in the field [30], which indicates genetic variation in resistance to herbivory.

We used the generalist hermaphroditic snail, *Arianta arbustorum* (Gastropoda: Helicidae), as the herbivore in our experiment. It occurs commonly throughout Europe [52], [53], and was found to feed on *L. flos-cuculi* in our study populations (D. Galeuchet, pers obs). *Arianta arbustorum* is also known to be an important herbivore of related plant species [54]. It is sensitive to several plant secondary compounds [55] and thus likely to respond to differences in plant resistance. To make sure that the snails used in our experiment would not be adapted to any of the plant origins, we collected them from a neutral environment, the Park Sanssouci in Potsdam, Germany.

### Plant material and experimental design

We used F2 plants from 13 to 19 (depending on the experiment) *L. flos-cuculi* populations located in calcareous fens in North-East Switzerland. Population sizes ranged from 40 individuals to ca 50000 individuals and the distances between the populations ranged from 1.2 to 68.9 km [30]. F2 plants were used, because outbreeding depression may not be detected in the F1 generation, but may appear only in later generations due to high heterosis [48], [56]–[59].

Initially, F1 offspring of within- and between-population crosses were obtained using 5 to 9 maternal plants from each population. Two flowers per individual were pollinated with a randomly selected plant from the same population and two with pollen of a randomly selected plant from one of the other populations. When the F1 plants were flowering, they were hand pollinated with other F1 plants (F1 × F1 crosses) to obtain the F2 plants. To obtain the F2 plants for within-population crosses each F1 plant resulting from a within-population cross was crossed with an unrelated F1 plant from the same population [60]. To obtain the F2 plants for between-population crosses each F1 plant resulting from a between-population cross was crossed with an unrelated F1 plant from the same between-population combination [60]. In the between-population outcrosses, different plants from each population received pollen from different randomly selected populations, which allows assessing general outbreeding effects. For our experiments we randomly selected seeds of the F2 families of the within- and between-population crosses from each of the populations and sowed the seeds in the greenhouse in February 2006. Seedlings were potted within few days and therefore they were approximately of the same age. The plants were grown in standard potting soil in 7×7 cm pots under natural light conditions and were well watered every day or every second day to correspond to the full-sun wet-grassland conditions in natural populations. Plant growth was not limited by nutrients or water. The treatments were conducted ca. three months after sowing when the flowering shoots started emerging, but plants had not started flowering yet. There were no signs of leaf senescence or turnover observed when the treatments were conducted.

To investigate resistance and tolerance to snail damage we repotted 2–16 seedlings per family (160 families in total). Half of the plants per family were randomly assigned to the herbivory treatment and the other half served as control. In the herbivory treatment, we covered the plants with cellophane bags, put one snail in each bag, and allowed it to feed on the plant for 6 days. The snails were collected in the field and starved for four days prior to the experiment and they were weighed immediately before and after use in the experiment. The control plants were bagged in the same manner as plants in the herbivore treatment. All snails were released after the experiment.

To investigate tolerance to artificial damage, i.e. to a combination of clipping and spraying with jasmonic acid, we repotted 2–16 seedlings per family (totally 149 families). We randomly assigned half of the plants per family to a clipping treatment where 50% of the rosette leaves were clipped and sprayed with jasmonic acid (250 mg jasmonate powder in 10 ml EtOH dilluted in 1.19 ml water and mixed up to 1.2 litres). The other half of the plants per family served as open control, in which the plants were not clipped and sprayed with 10 ml EtOH diluted in 1.19 ml water and mixed up to 1.2 litres. Since there were not enough plants to use replicates of exactly the same families for the snail herbivory and clipping treatments and their respective controls, we conducted and analyzed them separately. Before conducting the treatments, we counted the number of rosette leaves as a measure of initial plant size.

After the snail herbivory or clipping treatments the plants were allowed to grow in the greenhouse for another 8 weeks. By that time most plants were still flowering although many flowers had started to set fruits. Vegetative growth was still taking place and there were no signs of leaf senescence. We counted the number of rosette leaves as measure of plant size and counted the number of fruits produced.

### Measures of resistance and tolerance

Rausher [19] defined resistance as ‘any plant characteristic that influences the amount of damage a plant suffers from’. Plant resistance can be further measured in terms of antibiosis, which reduces herbivore performance [18], [60]. Therefore, we used two indirect measures of resistance, which have been commonly used in ecological and agricultural studies: resistance was determined as 1- the amount of leaf area consumed in terms of proportion of leaf area damaged by the herbivores (e.g., [42], [43], [45]) and inverse of herbivore performance on plants [22], [45]. In addition to defence chemistry, herbivore performance can also reflect the nutritive quality of plants, both of which can be influenced by the genetic background of plants.

To estimate resistance after the snails had been feeding on the plants we removed them and counted the number of damaged leaves and estimated the proportion of leaf area damaged visually to the nearest 1%. To aid estimating these percentages a transparent grid was placed on top of the leaves. Two persons estimated these damage percentages independently, which were then averaged for the analysis. We determined snail performance measured as final snail mass and corrected for differences in the initial mass in the statistical analyses. The damage levels in our experiment were similar to those observed in the field [15], [29], [60].

Plant tolerance to herbivory reflects the degree to which plants can re-grow after damage and can be investigated by comparing damaged and undamaged individuals or the impact of continuous damage on plant fitness from a group of related or clonally propagated plants (reviewed in [20]). We calculated and analyzed tolerance of each plant family separately for artificially damaged plants and for plants damaged by the snail herbivores. This was done, because tolerance can differ depending on the type of damage (e.g., [21]). To measure tolerance to artificial or snail damage we used the reaction-norm approach with two levels of damage for clipping (damaged and undamaged) and continuous levels of damage for snails [20], [21]. Confounding factors, such as environmental and genetic factors, that can influence natural damage levels may create statistical bias in tolerance measures [21], which is why we placed the snails to feed on each of the plants in the herbivory treatment instead of allowing them to choose freely the plants to feed on. Tolerance to artificial damage was assessed as the difference in fitness between undamaged and damaged plants of the same family and tolerance to snail damage was assessed as the slope of the proportion of leaf area damaged and number of fruits produced after damage [21]. There, negative slopes indicate that more damaged plants produce fewer fruits or fewer leaves after damage, i.e. poor tolerance. When there is no difference between damaged and undamaged plants, i.e. slope equals zero, plants are able to fully compensate damage. Positive slopes indicate very good tolerance or overcompensation (e.g., [20]).

### Statistical analyses

We conducted mixed-model analyses of covariance (ANCOVA) to test for differences in resistance between plants from within- and between-population crosses in the different populations. ANCOVA allows comparing series of regression models and thus to analyze data with both categorical and continuous explanatory variables [61]. We analysed two measures of resistance: 1-proportion of leaf damage and final snail mass (controlling for initial snail mass). In the latter measure, reduced herbivore performance indicates higher resistance or poorer plant quality for the herbivores. Population and family nested within population were used as random factors and cross was used as a fixed factor. Larger snails consumed greater amounts of leaves (*r* = 0.104, *p* = 0.0196). The relative growth rate of the snails was negatively correlated with their initial size (*r* = −0.345, *p* = 0.001). Plant size in terms of the number of rosette leaves was not correlated with snail mass or with their relative growth rate (r = 0.049, p = 0.2768; r = 0.005, p = 0.9043, respectively), which indicates that plant size has no impact on resistance or plant quality for the herbivores. Thus, initial snail mass was included as a covariate in the analyses of the proportion of leaf area damaged, number of damaged leaves, and the final snail mass, and initial number of rosette leaves was taken into account as a covariate in the analyses on the proportion of leaf area damaged. 1-proportion of leaf area damaged was log-transformed to meet the assumption of normal distribution.

To examine tolerance to artificial damage, we tested the effects of cross, population, family and clipping on the number of fruits produced by conducting a mixed model analysis of covariance. Treatment (clipped plus sprayed or not) and cross were used as fixed factors and population and family nested within population as random factors. Initial number of rosette leaves was used as a covariate in the analysis to account for differences in initial plant size.

Similarly, to examine tolerance to snail damage we conducted a mixed model analysis of covariance to test for the effects of cross and population and snail damage on the number of fruits produced. We treated cross as fixed factor and population and family nested within population as random factors. Adding the interaction terms of level of damage and population, cross and family nested within population allowed us to test the heterogeneity of the slopes (tolerance) and whether the slopes differ between the two crosses or among populations or families. The latter implies genetic variation in tolerance.

These analyses also served to test whether the effects of outbreeding on fitness are modified by herbivore damage, and whether the effects of herbivore damage on plant fitness depend on the cross and/or vary among populations. To examine outbreeding effects on fitness of bagged undamaged plants, we analysed the effect of cross, populations, and family also separately for these plants. Initial number of rosette leaves was taken into account as a covariate in all analyses of plant tolerance and fitness.

All analyses were conducted using the SAS software (version 9.1., SAS Institute, Cary, NC, USA). We determined appropriate error terms, degrees of freedom, and F-values for each analysis following Zar [62].
